# Solvent-Free Manufacturing of Electrodes for Lithium-ion Batteries

**DOI:** 10.1038/srep23150

**Published:** 2016-03-17

**Authors:** Brandon Ludwig, Zhangfeng Zheng, Wan Shou, Yan Wang, Heng Pan

**Affiliations:** 1 Department of Mechanical and Aerospace Engineering, Missouri University of Science and Technology, Rolla, MO-65409, US; 2 Department of Mechanical Engineering, Worcester Polytechnic Institute, Worcester, MA-01609, US.

## Abstract

Lithium ion battery electrodes were manufactured using a new, completely dry powder painting process. The solvents used for conventional slurry-cast electrodes have been completely removed. Thermal activation time has been greatly reduced due to the time and resource demanding solvent evaporation process needed with slurry-cast electrode manufacturing being replaced by a hot rolling process. It has been found that thermal activation time to induce mechanical bonding of the thermoplastic polymer to the remaining active electrode particles is only a few seconds. Removing the solvent and drying process allows large-scale Li-ion battery production to be more economically viable in markets such as automotive energy storage systems. By understanding the surface energies of various powders which govern the powder mixing and binder distribution, bonding tests of the dry-deposited particles onto the current collector show that the bonding strength is greater than slurry-cast electrodes, 148.8 kPa as compared to 84.3 kPa. Electrochemical tests show that the new electrodes outperform conventional slurry processed electrodes, which is due to different binder distribution.

Commercial Li-ion battery electrodes are manufactured by casting a slurry onto a metallic current collector. The slurry contains active material, conductive carbon, and binder in a solvent. The binder, most commonly polyvinylidene fluoride (PVDF), are pre-dissolved in the solvent, most commonly N-Methyl-2-pyrrolidone (NMP). During mixing, the polymer binder flows around and coat the active material and carbon particles^1^,^2^,^3^,^4^,^5^,^6^,^7^,^8^,^9^. After uniformly mixing, the resulting slurry is cast onto the current collector and must be dried. Evaporating the solvent to create a dry porous electrode is needed to fabricate the battery. Drying can take a wide range of time with some electrodes taking 12–24 hours at 120 °C to completely dry^5^,^10^. In commercial applications, an NMP recovery system must be in place during the drying process to recover evaporated NMP due to the high cost and potential pollution of NMP^11^,^12^. While the recovery system makes the entire process more economical it does require a large capital investment. Less expensive and environmentally friendly solvents, such as aqueous based slurries, could eliminate the large capital cost of the recovery system but the electrode would still require a time and energy demanding drying step^9^,^10^,^13^,^14^,^15^,^16^. Uncoventional manufacturing methods have also been used to create battery electrodes. Solvent based electrostatic spray deposition has been used to coat current collectors with electrode material^17^,^18^,^19^. This is achieved by adding high voltage to the deposition nozzle and grounding the current collector, which causes the deposition material to become atomized at the nozzle and drawn to the current collector. Electrodes constructed with this method exhibit similar characteristics as slurry-cast electrodes and have similar disadvantages in that they also require a time and energy intesive drying process (2 hours at 400 °C)^19^. Lithium ion batteries have also been manufactured using spray painting techniques by using NMP based paints to spray each electrode component onto the desired surface^20^. While this allows for battery fabrication on a variety of surfaces it still requires the solvent to be evoporated.

Electrodes manufactured with dry particles coated on current collectors represent the ideal manufacturing process, thereby eliminating solvents and the disadvantages that come with using them. Dry electrode manufacturing has been achieved through a variety of methods such as pulsed laser and sputtering deposition^21^,^22^,^23^,^24^,^25^,^26^,^27^. Pulsed-laser deposition is achieved by focusing a laser onto a target body containing the to-be-deposited material. Once the laser hits the target, the material is vaporized and deposited onto the collecting substrate. Although solvent is not used, the deposited film has to be subjected to very high temperatures (650–800 °C) to anneal the film^21^,^24^. Deposition via magnetron sputtering can lower the required annealing temperature to 350 °C^27^. While these methods are representatives of dry battery electrode manufacturing, they both suffer from very slow deposition rates and high temperature needs for annealing^19^.

A solution has been realized by using an electrostatic spraying system (Fig. 1A) to deposit completely dry electrode particles to the current collector (Fig. 1B). The process is commonly known as dry painting or electrostatic spraying. It consists of a powder pick-up and dispensing unit (such as Venturi pump) and an electrostatic spraying gun. A spraying gun is used to charge the fluidized dry particles. After being charged, the dry particles will be drawn to the ground current collector and deposited. A hot roller is used to control the electrode thickness and density (Fig. 1C,D) in place of the doctor blade typically used to control the thickness of a slurry-cast electrode^3^,^4^,^8^,^14^. Thermal activation of the binding material is quickly achieved using the hot roller, which takes the place of the oven needed to evaporate solvent in a slurry-cast electrode^1^,^3^,^5^,^16^. Dry painted electrodes exhibit good flexibility as well as the cability to coat very large areas (Fig. 1E).

Based on the cost analysis shown in Table 1, adopting the dry painting process can potentially save ~15% on labors, capital equipment and plant area in battery manufacturing. This is due to the removal of large capital investments such as solvent recovery systems and the removal of time and energy consuming drying processes needed for the conventional method of manufacturing. This analysis was based on the Argonne battery performance and cost (BatPaC) model^28^. In the BatPaC model, 7 different designs (different electrode sizes) with Manganese-spinel/Graphite chemistry were used to conduct the cost analysis. All designs show similar results, and we present the comparison between conventional slurry process with our proposed dry process for two designs (Design 1 and Design 4) here.

In addition, the dry painting process provides a possibility that the electrodes manufactured by this new method will have different microstructures than that of the conventional slurry-cast electrodes. With this consideration, in this work, electrodes manufactured using the new dry painting and slurry-cast process will be made with the same material composition (by weight percentage) and thickness. However, due to the distinct polymer binder dispersion processes involved, the electrodes fabricated by these two methods could exhibit different electrochemical properties, such as rate and cycling performance.

## Results

### Mechanical Bonding Characterization

Strong particle adhesion to the current collector is necessary when manufacturing battery electrodes, and ensuring a uniform distribution of binding material throughout the active and conductive particles is essential when trying to satisfy this requirement. Slurry-cast electrodes achieve even distribution of binding material by using solvent to dissolve the binding material, then mixing is performed to coat the remaining active and conductive particles. It could be assumed that the use of a solvent would allow the now liquefied binder to readily coat the remaining particles and diminish the need of an extensive mixing step, but this assumption would be incorrect. Extensive research has been conducted on the effects of the slurry mixing process on battery performance, with mixing anywhere from an hour to up to 3 days^1^,^16^. The mixing process is also crucial in achieving a high-strength electrode manufactured with the dry painting process.

The first tested dry painted electrodes were made by mixing as-shipped active (90% by weight), conductive (5% by weight), and binding material (5% by weight) together for 60 minutes in a high-energy mixer. LiCoO_2_ (LCO) was used as the active material, Super C65 Carbon (C65) as the conductive material, and PVDF for the binding material. After mixing, the powders were deposited onto the ground current collector (Al foil) using a high voltage electrostatic spraying gun. As-deposited electrodes were thermally activated on a hot plate set to 250 °C for 1 hour. A pull-off test was performed at the center of the coated area to evaluate the bonding strength of the particles to the current collector. The test results readily showed that the electrode had extremely low bonding strength (1.2 kPa) to the current collector as compared to a slurry-cast electrode (84.3 kPa) of similar composition.

**Figure 2:**
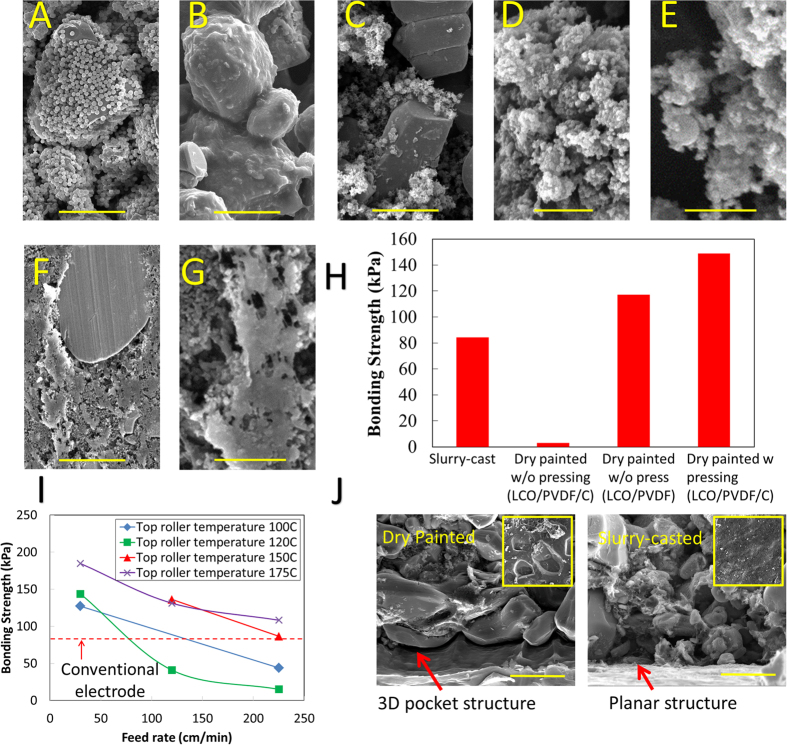
Mechanical Bonding Characterization. (**A**) SEM micrograph showing LCO covered with PVDF before thermal activation (scale bar is 5 μm). (**B**) SEM micrograph showing PVDF will completely wet the surface of LCO after thermal activation (scale bar is 5 μm). (**C**) SEM micrograph showing C65 Carbon will strip PVDF particles off LCO particles and subsequently form a layer around the PVDF particles (scale bar is 5 μm). (**D**) SEM micrograph showing what looks to be C65 agglomerations formed while mixing electrode materials for the dry painting process (scale bar is 5 μm). (**E**) SEM micrograph showing C65 is actually coating PVDF particles which is also the case for the previous image (**D**) (scale bar is 1 μm). (**F**) SEM micrograph a very flat top electrode surface due to the hot rolling process completed after deposition of the electrode material (scale bar is 5 μm). (**G**) SEM micrograph showing melted PVDF formed during the hot rolling process (scale bar is 1 μm). (**H**) Bonding strength (kPa) comparison of dry painted electrodes vs. slurry-cast electrodes. (**I**) Effects of top roller temperature and feed rate on mechanical strength of the electrodes. (**J**) SEM micrographs comparing the structure difference between dry and slurry-cast electrodes at the electrode – current collector interfaces (scale bar is 10 μm). Insets are top-view images of current collector after electrode failure.

A dry painted electrode made of 85% (by weight) LCO and 15% (by weight) PVDF (without C65) was tested to see if the bonding strength improved when only active and binding material is present. After thermal activation on a hot plate the sample was mechanically tested and found to have substantially higher bonding strength (117.1 kPa). It was concluded C65 had a detrimental effect on the bonding strength. An SEM micrograph (Fig. 2A) of the LCO/PVDF sample before thermal activation showed that LCO had a monolayer of PVDF particles covering it. After thermal activation the PVDF melts and wets the surface of LCO particles, creating contact points between surrounding LCO particles (Fig. 2B). This is a good indicator of strong bonding between particles and the bonding tests of this sample prove the strong bonding capability of dry painted electrodes.

An SEM micrograph of the first electrode (Fig. 2C) shows bare LCO particles and what could be assumed to be C65 agglomerations (Fig. 2D). Upon further inspection, it was discovered that the PVDF particles that once formed a uniform monolayer over LCO particles (Fig. 2A) have been completely stripped off LCO particles by C65 particles. The PVDF particles were subsequently covered with C65 particles. This was determined after examining what was thought to be C65 agglomerations more closely. It was found that the assumed to be C65 agglomerations (Fig. 2D) had a spherical shape comparable in size to raw PVDF particles. Furthermore, all SEM micrographs of this sample showed few uncovered PVDF particles even though 5% of the electrode was made of PVDF. Therefore, it was concluded that the PVDF particles have been largely coated with C65 particles. Clear evidence can be seen by few instances where PVDF is only partially covered by C65 (Fig. 2E). During thermal activation, the melted PVDF will be contained within the surrounding C65 particles. This will cause the LCO particles to remain loose without direct PVDF contact points. Thus, the electrode made with C65 had almost no bonding while the sample without C65 exhibited stronger bonding than the slurry-cast electrode.

A hot rolling step was introduced to the manufacturing process to simultaneously melt the PVDF particles and to press the neighboring particles together. Hot rolled electrodes exhibited a sharp increase in bonding performance (148.8 kPa) as compared to the original dry painted electrodes (1.2 kPa) and to the conventional slurry-cast process (84.3 kPa). It can be seen that the hot rolled electrodes are denser (Fig. 2F) than the original dry painted electrodes (Fig. 2C). The thermal budget (determined by feed rate and roller temperature) during the hot rolling process was enough to thermally activate the PVDF particles and create contact points between particles (Fig. 2G). A comparison of each of the tested manufacturing processes can be seen in Fig. 2H which shows the dry painted electrodes with the hot rolling step having the best bonding performance.

Further hot rolling tests were performed to study the effects the hot rolling temperature and hot rolling feed rates on the bonding performance of dry painted electrodes. The feed rate was set to three different values (30, 120, and 225 cm/min) while the top roller was set between 100 °C and 175 °C. The bottom roller was maintained constant at 190 °C to ensure at least one roller was set to a temperature about the PVDF melting temperature (177 °C). As expected, increasing the feed rate and reducing top roller temperature led to lower bonding strength due to a decreasing thermal budget (Fig. 2I). With top roller temperature at 150 °C or above, high feed rates (>120 cm/min) were allowed to produce electrodes with mechanical strength higher than conventional ones. It should be noted that all the pull-off tests fail at the electrode/current collector interface except for those with top roller temperature at 175 °C, which exhibited superior adhesion/cohesion strength of the electrode and fail due to current collector tearing. With lower top roller temperatures (120 °C or lower), the dependence of mechanical strength on temperature was not clear. In this temperature range, feed rate needs to be below 75 cm/min to ensure bonding strength comparable (or higher) than conventional ones.

It should also be noted that the conventional slurry-cast electrodes also failed at the electrode–current collector interface. The dry painted electrodes show stronger bonding (top roller temperature 100 °C and feed rate 30 cm/min) compared with conventional electrodes. Electrode–current collector interface tends to be the weaker due to the 2D planar contact nature. SEM micrographs (Fig. 2J) reveal pocket structures formed on current collectors resulting due to the mechanical pressing used in the dry process. This provides additional contact area compared with slurry process and renders additional adhesion strength for dry processed electrodes. Since all electrodes fail at the current collector interfaces in this study, it is unclear if dry electrodes yield higher cohesive strength within the electrode than conventional electrodes, which is subject to future studies.

The effect of compression ratio on mechanical strength was also performed. Electrodes with varying initial thickness were hot rolled to a final thickness of 50 μm and then mechanically tested (Fig. S1, in Supplementary Information). Bonding strength was practically non-existant for thinnest electrodes, but increased rapidly until a satisfactory strength (greater than or equal to the slurry tested electrodes) was reached with thicker electrodes (148.8 kPa).

### Electrochemical Characterization

**Figure 3:**
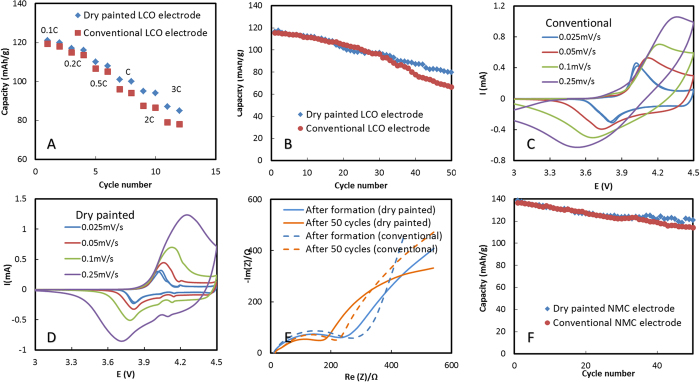
Electrochemical Characterization. (**A**) C-rate performance of the dry painted and conventional LiCoO_2_ (LCO) electrodes, (**B**) cycling performance comparison between the dry painted and conventional LCO electrodes; (**C**) Cyclic Voltammetry of conventional LCO electrodes; (**D**) Cyclic Voltammetry of dry painted LCO electrodes; (**E**) Comparison of electrochemical impedance spectra between dry and conventional LCO electrodes; (**F**) Cycling performance of the painted and conventional LiNi_1/3_Mn_1/3_Co_1/3_O_2_ (NMC) electrodes.

A direct comparison of electrochemical characteristics between dry painted electrodes and conventional slurry-casted electrodes has been performed. Both types of electrodes consist of 90% (by weight) LCO, 5% (by weight) carbon additive, and 5% (by weight) PVDF. The composition was selected to maximize the energy density while maintaining sufficient electron conductivity and mechanical integrity. The dry painted (after hot rolling) electrode has a free-standing porosity around 30%, while the conventional cast electrode porosity is about 50%. The conventional electrode was also pressed to around 30% for direct comparison with dry electrodes. The porosity measurement is described in Methods. Figure 3A shows the rate performance of the dry painted LCO electrodes at various discharge currents ranging from 0.1–3 C along with conventional slurry-cast electrodes. For the dry painted electrodes, the cell delivers a specific capacity of 121 mAhg^−1^ at 0.1 C, 89% of theoretical capacity (the theoretical capacity is 137 mAhg^−1^ for LCO over the voltage range 4.2–2.5 V vs. Li/Li^+^ because at the charge cut-off, 4.2 V, LCO is partially delithiated to Li_0.5_CoO_2_). At 0.2 C, 0.5 C, 1 C, 2 C and 3 C, the capacity lowered to 117 mAhg^−1^, 110 mAhg^−1^, 101 mAhg^−1^, 95 mAhg^−1^, and 87 mAhg^−1^, which are 86%, 80%, and 74%, 70%, and 64% of the theoretical capacity, respectively. Overall, the dry printed electrode has higher capacity than the conventional slurry-cast electrodes (Fig. 3A).

The cycling performance of the dry painted and conventional LCO electrode is shown in Fig. 3B. For the painted electrode, the discharge capacity versus corresponding cycle number decays from 114 mAhg^−1^ in the initial cycle to 80 mAhg^−1^ after 50 charge/discharge cycles, 70% capacity retention at 0.5 C after 50 cycles. For the conventional electrode, after 50 cycles, only 58% capacity is retained. The painted electrode has higher cycling stability than the conventional electrodes (Fig. 3B).

To understand the mechanism that allows the dry painted electrodes to outperform the conventional electrodes, both electrodes were examined by Cyclic Voltammetry (CV) and electrochemical impedance spectra (EIS). Figure 3C,D compare Cyclic Voltammograms of the painted and conventional LCO electrodes. At a scan rate of 0.025 mV/s, a single pair of oxidation and reduction peaks, the reduction peak at ~3.8 V and the oxidation peak at ~4 V corresponding to a Co^3+^/Co^4+^ redox couple, is observed for both electrodes, indicating the good reversibility of lithium insertion into and extraction from LCO. With the increased scan rate, the painted electrodes largely maintain the symmetrical shape of the cathodic peaks and the anodic peaks in their CV curves, whereas the shapes of the cathodic peaks and the anodic peaks change significantly for the conventional electrodes. Moreover, the potential difference between the cathodic peak and the anodic peak at a certain scan rate in the painted electrode is smaller than that in the conventional one, indicating that the painted electrode has lower electrochemical polarization and better rate capability.

Nyquist plots of the painted and conventional LCO electrode/Li cell at fully discharged state are shown in Fig. 3E. Impedance is a collective response of kinetic processes with different time regimes. All the plots consist of an intercept with the Re(Z) axis, a high-frequency semicircle and a low-frequency tail. The intercept with the Re(Z) axis at high frequency referes to the total amount of Ohmic resistance, including electrolyte resistance and electric contact resistance. This resistance is much smaller than the other contributions of resistance. The semicircle can be attributed to the electrode-electrolyte interfacial impedance, while the tail attributed to the diffusion-controlled Warburg impedance. Both electrodes show slightly decrease in interfacial impedance with cycles. The width of the semicircle of the painted electrode is smaller than that of the conventional one, indicating that the dry painted electrode has slightly lower interfacial resistance. After cycling, the width of the semicircle of the painted electrode is still smaller than that of the conventional one.

To prove its versatility of the dry manufacturing process, LiNi_1/3_Mn_1/3_Co_1/3_O_2_ (NMC) electrodes were also manufactured. The cycling performance of the painted and conventional NMC electrodes is shown in Fig. 3F. For the painted electrodes, the discharge capacity versus corresponding cycle number decays from 138 mAhg^−1^ in the initial cycle to 121 mAhg^−1^ after 50 charge/discharge cycles in the voltage of 2.8–4.3 V, meaning that there is 87% capacity retention at 0.5 C after 50 cycles. For the conventional electrodes, after 50 cycles, 84% capacity is retained. The painted electrodes have slightly better cyclability than the conventional ones. Other electrochemical characterizations, including the C-rate performance and CV comparisons, indicate dry painted NMC electrodes slightly outperform the conventional ones (Fig. S2, Supplementary Information).

## Discussion

SEM micrographs (Fig. 2A) showed a tendency for PVDF to attach and coat LCO particles without C65. When C65 is mixed in, the PVDF is stripped off of the LCO particles and readily coated by C65 particles (Fig. 2C). To understand this mixing behavior, surface energy measurements were conducted for LCO, C65, and PVDF to help explain the results of the mixing process and to help predict the mixing characteristics of various electrode materials.

**Figure 4:**
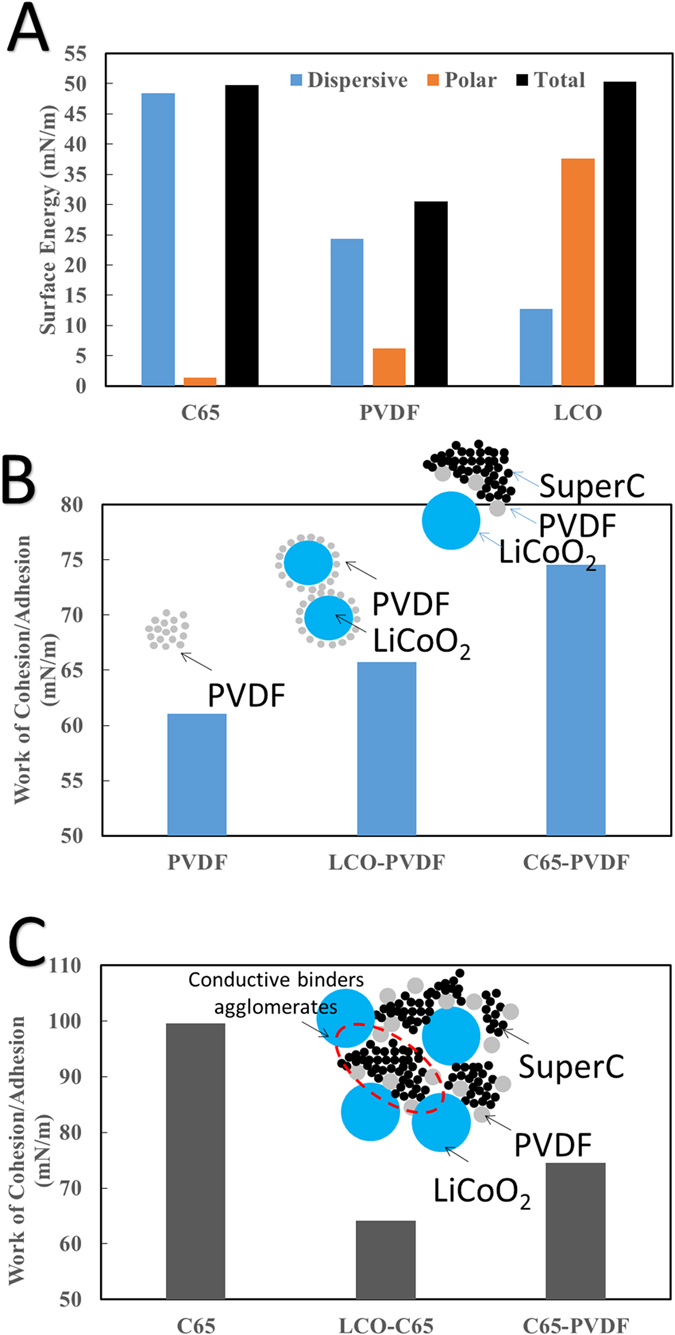
Material Surface Energy Characterization. (**A**) Dispersive, polar, and total surface energy values calculated using the Sessile drop contact angle method. (**B**) Work of cohesion for PVDF and also the work of adhesion for PVDF-C65 and LCO-PVDF, which suggest PVDF will preferably form on C65. (**C**) Work of cohesion for C65 and also the work of adhesion for LCO-C65 and C65-PVDF, which suggests C65 particles will preferably stick to one another.

The sessile drop contact angle method (Fig. S3–S5, Supplementary Information) was used to determine the polar and dispersive surface energy components for each of the materials used (Fig. 4A). LCO shows a strong polar component (37.57 mN/m) and a low relatively low dispersive component (12.75 mN/m). C65 shows opposite surface energy characteristics with it having a very large dispersive component (56.27 mN/m) and an almost non-existent polar component (0.54 mN/m). Polar and dispersive surface energy components for PVDF have values located between the respective values of LCO and C65.

With LCO and C65 having extreme polar and dispersive components, they were found to heavily impact the distribution of PVDF throughout the composite. Using measured surface energy, the work of adhesion (cohesion) between two (single) materials can be calculated by Fowkes equation,


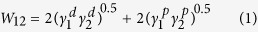


where *γ*_*1*_^*d*^ and *γ*_*2*_^*d*^ are the dispersive surface energies of material 1 and 2 while *γ*_*1*_^*p*^ and *γ*_*2*_^*p*^ are the polar surface energies of material 1 and 2^29^. The work of adhesion calculated for PVDF to LCO and C65 show that they are higher than the work of cohesion for PVDF-PVDF contacts (Fig. 4B). This result shows that PVDF will more readily attach to LCO or C65 when either is present than to form PVDF agglomerations. The preferential adhesion of PVDF to LCO is desirable and will facilitate more even distribution throughout LCO particles and help increase the bonding performance. It should be noted that the work of adhesion between PVDF and C65 is stronger than that of PVDF and LCO. This helps to explain the observations in SEM micrographs (Fig. 2) where PVDF was shown to readily coat LCO particles but were subsequently stripped off and covered when C65 was introduced to the mixture.

Work of adhesion calculations for C65 to LCO and PVDF show that C65 will preferably attach to C65 itself and form agglomerates, Fig. 4C. Since adhesion between C65-PVDF is comparable to C65-C65, PVDF will be intermingled with C65 and form agglomerates, which will be referred to as “conductive binder agglomerates”, as shown in insert of Fig. 4C. Due to the weaker interactions of either C65 or PVDF with LCO, the “conductive binder” largely maintains its agglomeration form and merely distributes around LCO particles, as illustrated in Fig. 4C. This unique distribution, as reasoned from surface energy analysis, has also been verified by SEM micrographs which show the distributions of C65/binder agglomerates when mixed with LCO (Fig. 2C,D).

Furthermore, the measured surface energies can provide insight into the wetting behavior of melted PVDF particles. Using the Fowkes equation^29^,


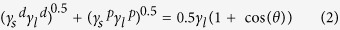


where subscript *s* and *l* represent LCO and PVDF, superscripts *d* and *p* represent dispersive and polar components, and Θ is the contact angle. Using the surface energy components previously found for LCO and PVDF, the calculation shows that PVDF will completely wet LCO surface upon melting. Therefore, full coverage of PVDF on LCO can be expected which agrees with SEM images (Fig. 2B). Certainly, with the presence of C65, the wetting of PVDF on LCO will be hindered.

The different manufacturing processes will result in different binder distributions and hence the electromechanical properties of the electrodes will vary. In the porous electrode composite, ions move through the liquid electrolyte that fills the pores of the composite. Electrons are conducted via chains of carbon particles through the composite to the current collector. PVDF holds together the active material particles and carbon additive particles into a cohesive, electronically conductive film, and provide the adhesion between the film and the current collector. It is well known that when it is in contact with the surface of particles, a polymer tends to chemically bond or physically absorb to form a bound polymer layer on the surface of the particles of active material and carbon additive, and polymer chains tend to aligning with the surface^7^,^8^. This bound polymer layer can interact with adjacent polymer layer to form the immobilized polymer layers due to reduced mobility. Bound and immobilized layers together are considered as fixed polymer layers^9^. Following the formation of fixed polymer layers on particle surfaces, free polymer domains start to appear^8^. The free binder polymers are crucial to the mechanical strength of the electrodes. Due to the substantially large surface area of active material and carbon additive present in electrodes, almost all of binder polymers are in the fixed state, and very limited polymers are free^9^. Therefore, for a given electrode manufacturing method, the electrode composition and binder distribution has a significant effect on electrochemical properties.

**Figure 5:**
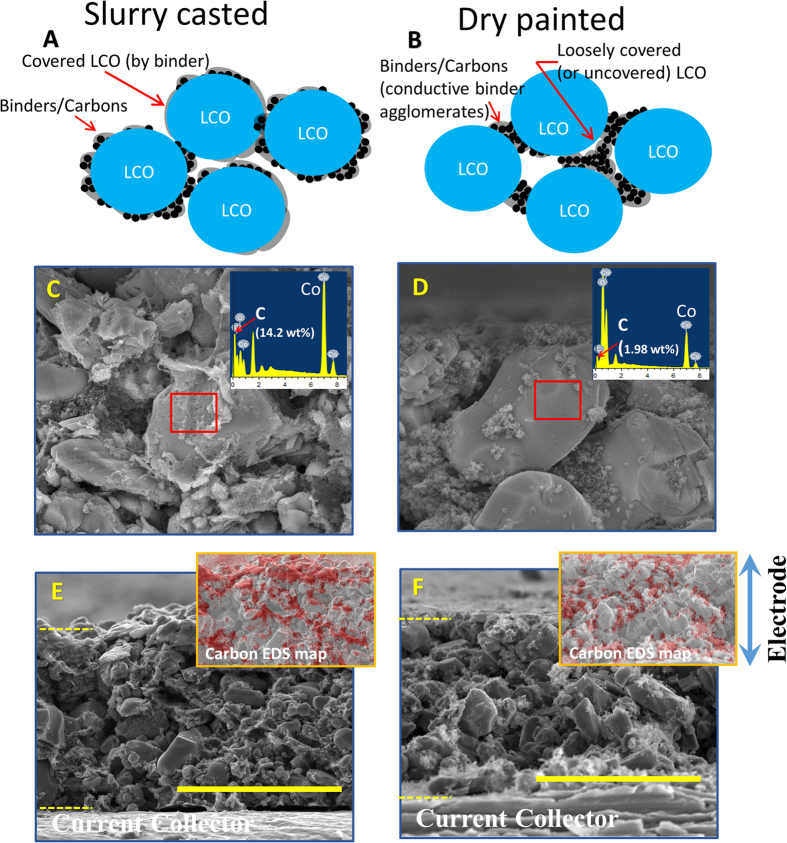
Electrode Cross-section Charecterization. Schematic illustrations of characteristic binders/carbons distribution in dry painted electrodes (**A**) and conventional electrodes (**B**). SEM micrograph showing the representative LCO particles in cross-sectioned dry painted electrodes (**C**) and conventional electrodes (**D**). SEM showing cross-section of a 90% LCO, 5% C65, 5% PVDF dry painted electrode and conventional electrodes (**F**) (scale bar is 50 μm). Insets in (**E**,**F**) are EDS (Energy-dispersive X-ray spectroscopy) mapping of carbon elements (colored red) on the entire electrodes.

It is believed that the lower interfacial resistance in dry painted electrodes likely results from the difference in binder distributions induced by different manufacturing methods. In the conventional method, PVDF is dissolved in NMP solvent followed by a prolonged drying process. Dissolved binders form thin carbon/binder layer extensively covering LCO particles after solvent evaporation (Fig. 5A). As a semi-crystalline polymer, PVDF tends to form crystallite regions upon drying, leading to the formation of fixed polymer layers^10^. In the dry process, on the contrary, binders and carbon are mixed to form the “conductive binder agglomerates” around LCO particles. Due to the presence of carbon, the extended wetting and spreading of binder on LCO surface are greatly hindered. Only near the necking area between two neighboring LCO particles are the binders in the “conductive binder agglomerates” forced to wet and bond to LCO surface during the hot rolling process. On the locations away from the necking area, binders only loosely bond to LCO surface (Fig. 5B). As a result, cross-sectional SEM images show more “un-covered” LCO particles in dry electrodes (Fig. 5D), while conventional electrodes exhibit mostly covered LCO particles (Fig. 5C). Moreover, the Energy-Dispersive X-ray spectra (EDS) in selected areas of both electrodes reveal the covering layer primarily consists of carbon (carbons and/or binders). Detailed SEM/EDS analysis confirming the binder coverage in the two types of electrodes can be found in Supplementary Information (Fig. S6). Thus, it is concluded that the dry process results in less fixed polymer layers on LCO surfaces, which contributes to lower interfacial impedance. Lithium ions will easily diffuse into/out of the LCO particles without PVDF coverage for the dry painted electrodes. It should be emphasized that the “conductive binder agglomerates” largely fill the space between LCO particles, as can be verified by the SEM images (with inserted EDS maps of carbon) in Fig. 5E,F showing conductive paths formed at the gaps between LCO particles throughout the entire electrode.

## Conclusion

The results prove that Li-ion battery cathodes can be manufactured using a completely dry material coating process, which paves the way for a more efficient and fast battery manufacturing method. The new dry manufacturing method integrates electrostatic spray and hot rolling processes to realize materials dispensing and binder activation. The dry manufacturing method can be easily implemented on existing roll-to-roll battery production line. The mechanical strength and electrochemical performance of dry manufactured electrodes slightly outperform conventional ones due to unique binder distribution observed in dry manufacturing process. Both dry painted LCO and NMC electrodes have been fabricated, which clearly shows the versatility of the dry painting technology.

## Methods

### Cathode Powder Preparation

Dry cathode materials, LCO (MTI), Super C65 Carbon Black (Timcal), and PVDF (MTI), were mixed with zirconia beads in a BeadBug Microtube Homogenizer (Benchmark Scientific) for 30 minutes at 2800 RPM. For cathodes made with NMC (Umicore), the same mixing parameters were used. The details on the mixing and mixing time effects can be found in Fig. S7 Supplementary Information.

### Material Deposition

After mixing, the powders were added to fluidized bed spraying chamber. The fluidzed bed chamber was fed into the spraying system with the electrostatic voltage set to 25 kV while the carrier gas inlet pressure was set to 20 psi. Distance from the deposition head to the grounded aluminum current collector was kept constant at 1.5 in. Surface morphology of the deposited material was investigated using a Helios NanoLab DualBeam operating with an emission current of 11 pA and 5 kV accelerating voltage. The details in spraying setup configuration, thickness control and material composition on spraying behaviors can be found in Fig. S8 Supplementary Information.

### Porosity Measurement

Porosity of the sprayed (or cast) electrode was determined by taking into account of the theoretical density of the mix (active material, carbon black, and binder) according to the following equation^7^.


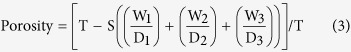


where T is the thickness of the electrode laminate (without Al foil current collector), S is the weight of the laminate per area, W_1_, W_2_, and W_3_ are the weight percentage of active material, PVDF binder and C65 within the electrode laminate, while D_1_, D_2_, and D_3_ are the true density for LCO (or Li[Ni_1/3_Co_1/3_Mn_1/3_]O_2_), PVDF and C65, respectively. The theoretical densities for LCO (or NMC) active material, PVDF and C65 are 5.1 (or 4.68), 1.78, and 2.25 g cm^−3^, respectively. All the porosities were calculated by assuming that the weight fractions and density of each material were not changed by the fabrication process. In general, electrodes with porosity about 30% have good electrochemical performance.

### Mechanical Bonding Measurements

For early mechanical bonding test, the coated current collectors were placed onto a hot plate for 1 hour at 250 °C. A hot roller was used for thermal activation and increasing the density of the electrode material. The bottom roller temperature was set to 190 °C and the top roller temperature varied from 100 to 175 °C. Feeding rates of 30, 120, and 225 cm/min. were used. A Mark-10 Series 4 force gauge was paired with a Mark-10 ES10 manual hand wheel test stand to determine the bonding strength of the coated electrode material. To test the stength, the coated current collector was mounted onto the test stand base with the center of the coated region directly below the force gauge. A 0.5 in. diameter flat head (Mark-10) was attached to the force gauge with a piece of double sided tape (7 mm by 12 mm) attached to the flat head. The force gauge was lowered until the flat head touched the substrate and compressed to 50 N. After compression, the force gauge was raised at a rate of 1 rotation over 20 seconds until the tape attached to the flat head decoupled from the coated area. The maximum tensile force was recorded and converted to the maximum strength by incorporating the known contact area of the tape.

### Electrochemical Measurements

Dry sprayed electrodes were electrochemically tested against Li foil in a Swagelok cell with stainless-steel current collectors. A collector was covered with a piece of Li foil and two pieces of Celgard 2500 microporous separator were placed over the Li foil. A piece of the cathode material was then centered over the separator and the cell was sealed to ensure good contact between the cathode and the other collector. 1 M LiPF6 in ethylene carbonate (EC), diethyl carbonate (DEC), and dimethyl carbonate (DMC) (1:1:1) was used to as electrolyte to fabricate the cells. Each cell was tested with a galvanostat/potentiostat/impedence analyzer (Bio-logic VMP3). For rate performance, cells were charged to 4.2 V and discharged to 2.5 V at various rates such as 0.1 C, 0.2 C, 0.5 C, 1 C, 2 C, 3 C and 5 C. For cycling performance, cells, were charged to 4.2 V and discharged to 2.5 V at 0.5 C. Constant current charging and discharging were used for all tests. Electrochemical impedance spectroscopy measurements were carried out from 0.1 Hz to 200 KHz using a 10 mV AC signal.

## Additional Information

**How to cite this article**: Ludwig, B. *et al.* Solvent-Free Manufacturing of Electrodes for Lithium-ion Batteries. *Sci. Rep.* **6**, 23150; doi: 10.1038/srep23150 (2016).

## Supplementary Material

Supplementary Information

## Figures and Tables

**Figure 1:**
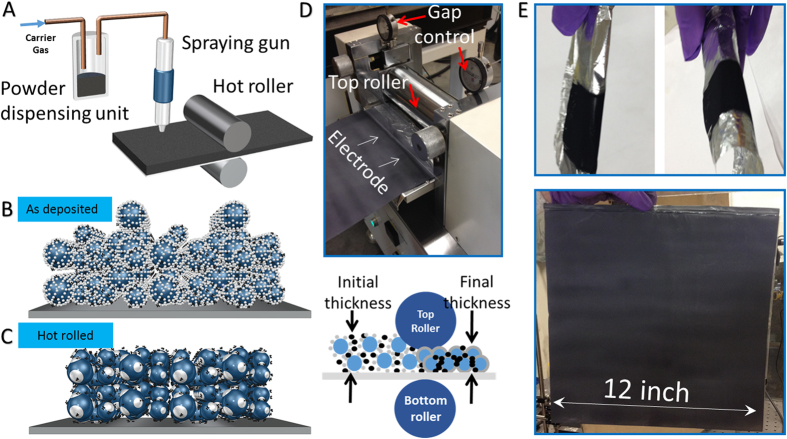
Dry Painted Battery Concept. (**A**) Manufacturing system for electrodes created by dry particle painting process. (**B**) 3D representation of a dry painted electrode before thermal activation. (**C**) 3D representation of a dry painted electrode after hot rolling and thermal activation. (**D**) Hot roller configuration. (**E**) Dry painted electrodes on Al foils.

**Table 1:** Cost analysis of conventional slurry process with our proposed dry process*.

	Battery Design 1			Battery Design 4		
	Direct Labor,hours/year	Capital equipment,Millions	Plant area,Square meters	Direct Labor,hours/year	Capital equipment,Millions	Plant area,Square meters
Conventional process	511,871	109.85	12,569	595,918	139.10	15,958
Dry process	441,021	94.28	10,918	499,600	112.61	13,326
Saving percentage	21.6%	14.2%	13.1%	16.2%	19.0%	16.5%

^*^The number of battery packs manufactured per year is assumed to be 100,000.
