# Supplementary material for: Solvent-Free Manufacturing of Electrodes for Lithium-ion Batteries
Source: Sci Rep. 2016 Mar 17;6:23150. doi: 10.1038/srep23150 (PMC4794730; doi:10.1038/srep23150)
Supplement: Supplementary Information [file srep23150-s1.docx]

Supplementary Information

**Solvent-Free Manufacturing of Electrodes for Lithium-ion Batteries**

Brandon Ludwig^1^, Zhangfeng Zheng^2^, Wan Shou^1^, Yan Wang^2^, Heng Pan^1^

^1^Department of Mechanical and Aerospace Engineering, Missouri University of Science and Technology, Rolla, MO-65409

^2^Department of Mechanical Engineering, Worcester Polytechnic Institute, Worcester, MA-01609

**Table of Contents**

- Effect of Compression Ratio on Mechanical Strength during Hot Rolling
- Electrochemical Characterization of NMC Electrodes
- Surface Energy Measurement and Discussions
- Binder Distribution Difference between Dry Painted and Slurry-casted Electrodes
- Effect of Mixing Time
- Effect of Spraying System Setup Parameters
- Control of Electrode Thickness

**Effect of Compression Ratio on Mechanical Strength**

The mechanical (bonding) strength of the electrode is critically determined by the roll pressure applied during hot rolling process. Roll pressure can be related to compression ratio, (ratio of intial thickness to final thickness) by $p_{r}=\frac{F_{rv}}{wl}$ (1)

where *F_rv_* is the radial load, and *wl* is the product of the electrode width and projected length of the contact area^1^. The width is kept constant for all electrodes while the projected length of the contact area is dependent on the compression ratio given by

$l\approx{[R\left( \frac{h_{1}}{h_{2}}-1 \right)]}^{0.5}$ (2)

where *R* is the radius of the rollers, *h_1_* is the initial electrode thickness, and *h_2_* is the final electrode thickness. The effect of the compression ratio on the bonding strength capabilities of dry-painted electrodes was found by mechanically testing electrode samples with varying initial thicknesses. Each of the electrodes were hot rolled with a set roller gap. It is found that the bonding strength increases drastically when the initial thickness increases is 1.5 to 2.5 times that of the final electrode thickness. A maximum mechanical strength of 153.09 kPa was found when the compression ratio was 6.5 but a marginally lower strength of 134.29 kPa was found when the compression ratio was 2.75 (Fig. S1). Large compression ratio were found due to the extremely porous nature of the unpressed electrodes but large initial thickness values will evetually lead to an undesired final thickness due to the inability of the roller system to further press the powders.

**Supplementary Figure 1 | Effect of Pressing Ratio on Mechanical Strength.**

**Electrochemical Characterization of NMC Electrodes**

To prove its versatility, LiNi_1/3_Mn_1/3_Co_1/3_O_2_ (NMC) NMC electrodes were also manufactured by the dry process. The effect of C-rate on the electrode performance was investigated by discharging at different rates, from 0.1C to 3C. For comparison, a conventional NMC electrode was also included. The effect is shown in Fig. S2(a). It is clear that the painted NMC electrode has better rate performance than the conventional one. Figure S2(b) shows cyclic voltammograms (CV) at a scan rate of 0.05mV/s. Both electrodes have only one anodic peak and one cathodic one, which can be possibly identified as Ni^2+^/Ni^4+^ redox couple. The single anodic peak indicates that Ni^2+^/Ni^4+^ oxidization is direct, not via Ni^3+^. Indirect oxidation would exhibit two anodic peaks in the CV curves, denoted as Ni^2+^/Ni^3+^ and Ni^3+^/Ni^4+^ processes. This could be due to the Jahn-Teller distortion of Ni^3+^ (d^7^) in NiO_6_ octahedra resulting in the direct oxidation of Ni^2+^ to Ni^4+ 2,3^. It is evident that the potential difference between the cathodic peak and anodic peak in the painted NMC electrode is also smaller than that in the conventional one, as observed in LCO electrodes.


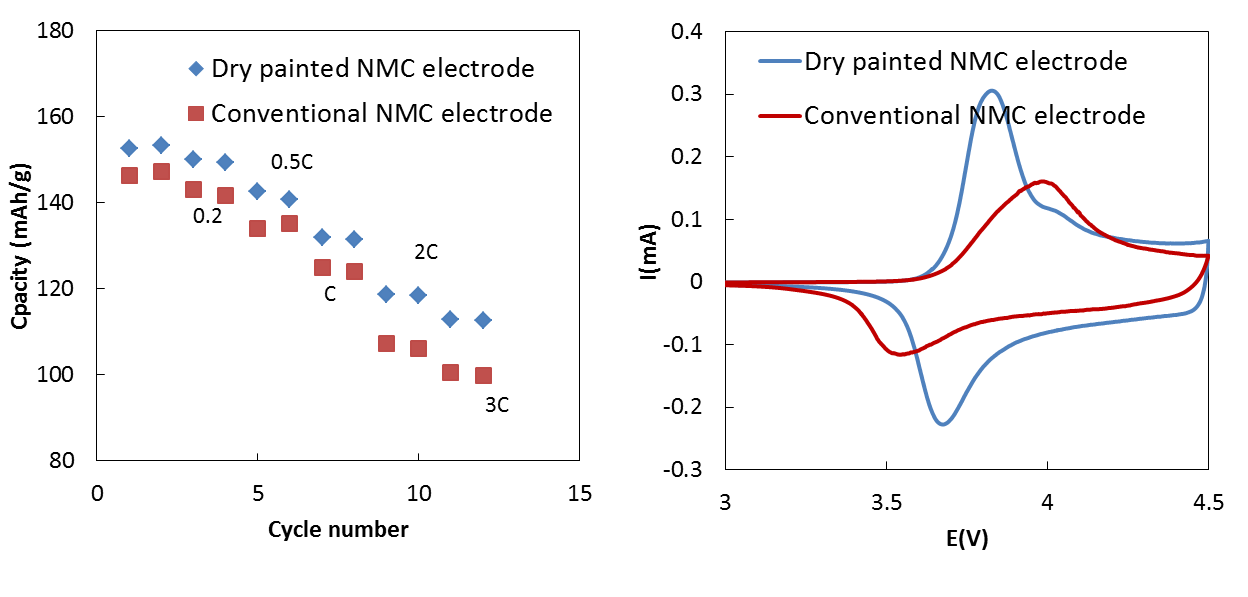


B

A

**Supplementary Figure 2 | Electrochemical Characterizaiton of NMC Electrodes.** a) C-rate performance of the dry painted and conventional NMC electrodes, (b) Cyclic Voltammetry of dry painted and conventional NMC electrodes.

**Surface Energy Measurement and Discussions**

In this work, the surface energy of the used electrode powders is needed. The Sessile Drop Technique was used as the characterization method. The Sessile Drop Technique is a contact angle method used to measure the contact angle of a selected liquid dropped on to a flat solid material. Typically, this method is used to measure the surface energy characteristics of an unknown material by relating the known surface energy components of the liquid to the measured contact angle of the droplet formed on the unknown surface. In most cases this method is used for solid bulk materials where the problems associated with the used liquid soaking into the substrate are not relevant.

An OCA15EC/B Contact Angle Measuring Device by DataPhysics was used to capture the contact angles and to calculate polar, dispersive, and total surface energies of the battery electrode materials. As previously mentioned, the Sessile Drop Technique is typically performed on a flat bulk solid material; therefore, packing each electrode powder as densly as possible is required. This will allow the densely packed particles to be analyzed as a single solid material^4^. If the powders are not packed well the probling liquid will readily soak through the pores between the particles. LiCoO_2_ (LCO), polyvinylidene fluoride (PVDF), and Super C65 Carbon Black (C65) were the three materials tested and each required their own set of packing requirements.

LCO was characterized with an LCO sputtering target from Kurt J. Lesker Company. The target provides a highly pure (99.7%) and dense material to perform the surface energy characterization. Four probing liquids were used for characterizing LCO, which include dimethyl sulfoxide (DMSO), formamide, thiodiglycol (TDG), deionized water. The results show that the droplets form contact angles of 35.65° for DMSO (Fig. S3(a)), 49.55° for formamide (Fig. S3(b)), 39.83° for TDG (Fig. S3(c)), and 48.13° for water (Fig. S3(d)).

Each of the contact angles were entered into the software provided by DataPhysics to determine the polar, dispersive, and total surface energy components for LCO. The Owens, Wendt, Rabel, and Kaelble (OWRK) method for calculated surface free energy was used characterize LCO. It was found that the surface energy due to polar interactions was 37.57 mN/m while the surface energy due to dispersive interactions was 12.75 mN/m. A line fitted to the plotted points for each of the probing liquids used in the OWRK method gave an error value of 0.9938 and it can be seen that the fitted line represents a very close fit to the plotted points (Fig. S3(e)). The surface energy values obtained from this approach were used for future analysis involving LCO.


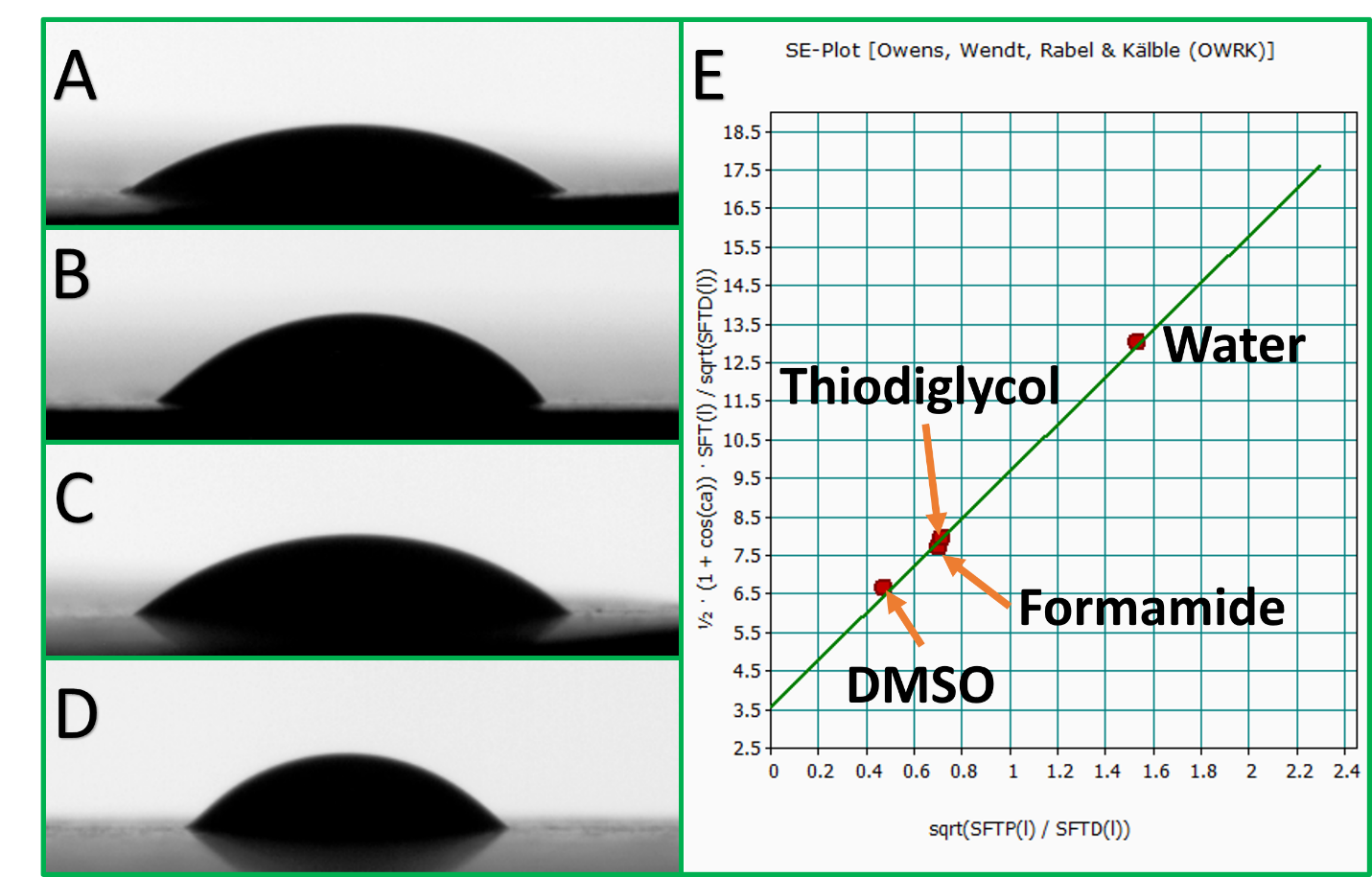

**Supplementary Figure 3** **| LCO Surface Energy Characterization.** Droplets formed by a) DMSO, b) formamide, c) TDG, and d) water on LCO to determine the contact angles needed for surface energy characterization for LCO. e) Surface energy plot for each of the probing liquids used to estimate the surface free energy components of LCO.

The PVDF sample used for surface energy characterization was made in the laboratory. As-shipped PVDF was pressed into a 12.5 mm diameter disc-shaped pellet by adding PVDF powder into a pressing die and pressing. A pressing pressure of 263 MPa was used and the pressing process was completed using a Carver Bench Top Press. Three fluids were used to characterize the PVDF: ethylene glycol (EG), formamide, and TDG. The EG droplet formed a contact angle of 57.12° (Fig. S4(a)) while formamide and TDG droplets formed contact angles of 64.40° (Fig. S4(b)) and 59.97° (Fig. S4(c)), respectively.

Similarly to LCO, the contact angles for each of the probing liquids were entered into the surface energy software and the polar, dispersive and total surface energy components for PVDF were calculated. The OWRK method was used for the calculation and returned a polar surface energy component of 6.18 mN/m and dispersive surface energy component of 24.33 mN/m. Again, a fitted line to the plotted points (Fig. S4(d)) shows a very close fit (error value of 0.9999). Given the very close fit the obtained surface energy components were used for future analysis involving PVDF.


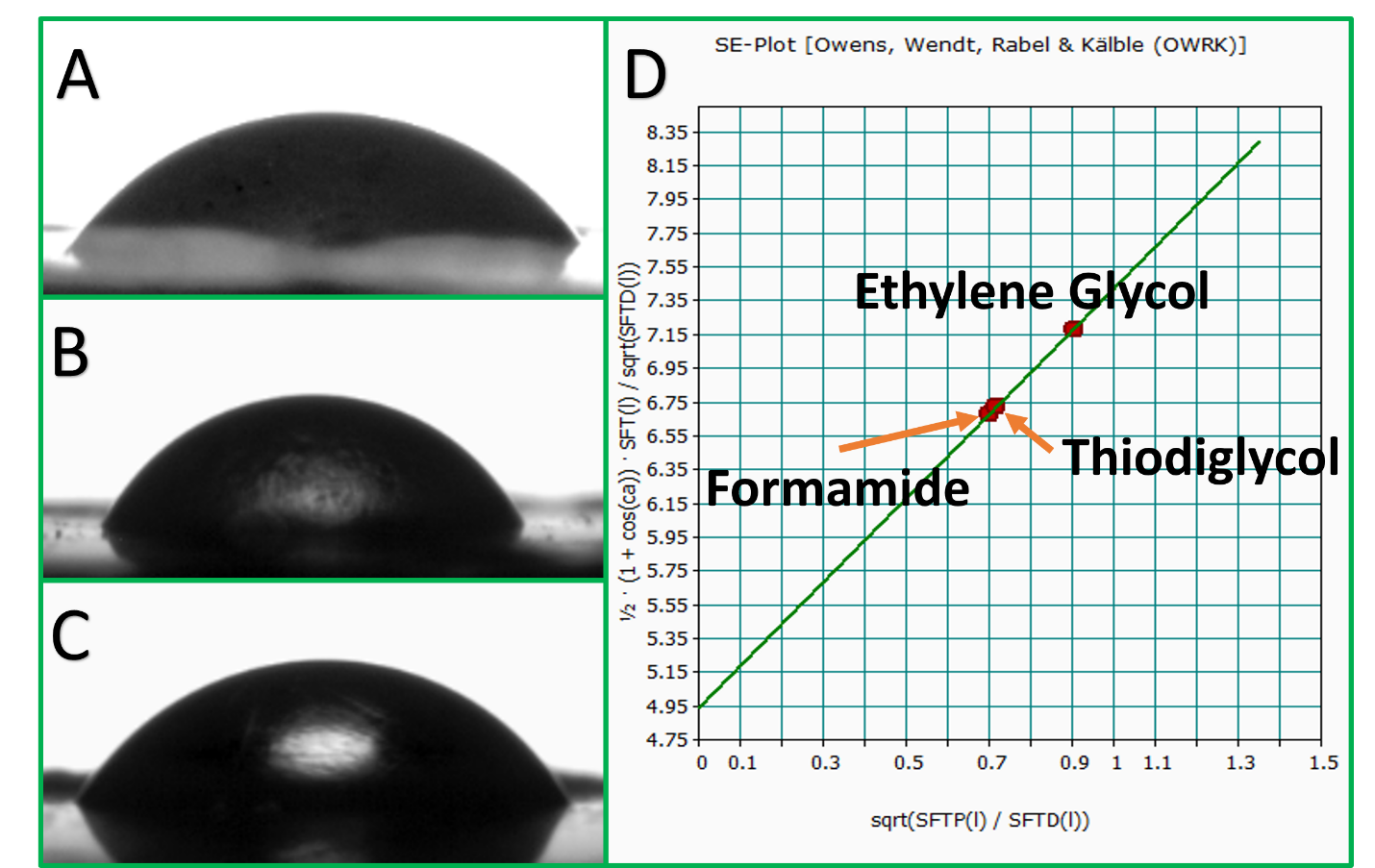


**Supplementary Figure 4 | PVDF Surface Energy Characterization.** Droplets formed by a) EG, b) formamide, and c) TDG on PVDF to determine the contact angles needed for surface energy characterization for PVDF. d) Surface energy plot for each of the probing liquids used to estimate the surface free energy components of PVDF.

Polar and dispersive surface energy measurements using the Sessile Drop Technique proved to be most difficult for C65. For C65, the small particle size leads to many pores throughout the packed material. When the probing liquid was dropped onto the surface it was quickly absorbed by the packed material, making the measurement of the contact angle extremely difficult^4^. As a result, conductive graphite powder (MTI Corp.) was used due to its planar structure allowing it to be more easily pressed. A graphite pellet was pressed similarly to the PVDF pellet previously discussed. EG, formamide, and TDG were used as the probing liquids to characterize graphite. The EG droplet formed a contact angle of 60.06° (Fig. S5(a)) while formamide and TDG droplets formed contact angles of 79.82° (Fig. S5(b)) and 75.05° (Fig. S5(c)), respectively.

Upon entering the respective contact angles for each of the probing liquids into the surface energy software, the polar, dispersive, and total surface energy components for graphite were found. The OWRK method shows a polar component of 0.54 mN/m and dispersive component of 56.27 mN/m. A fitted line to the plotted probing liquid points again show a very good fit (Fig. S5(d)); therefore, the surface energy values obtained were used for future analysis involving C65.


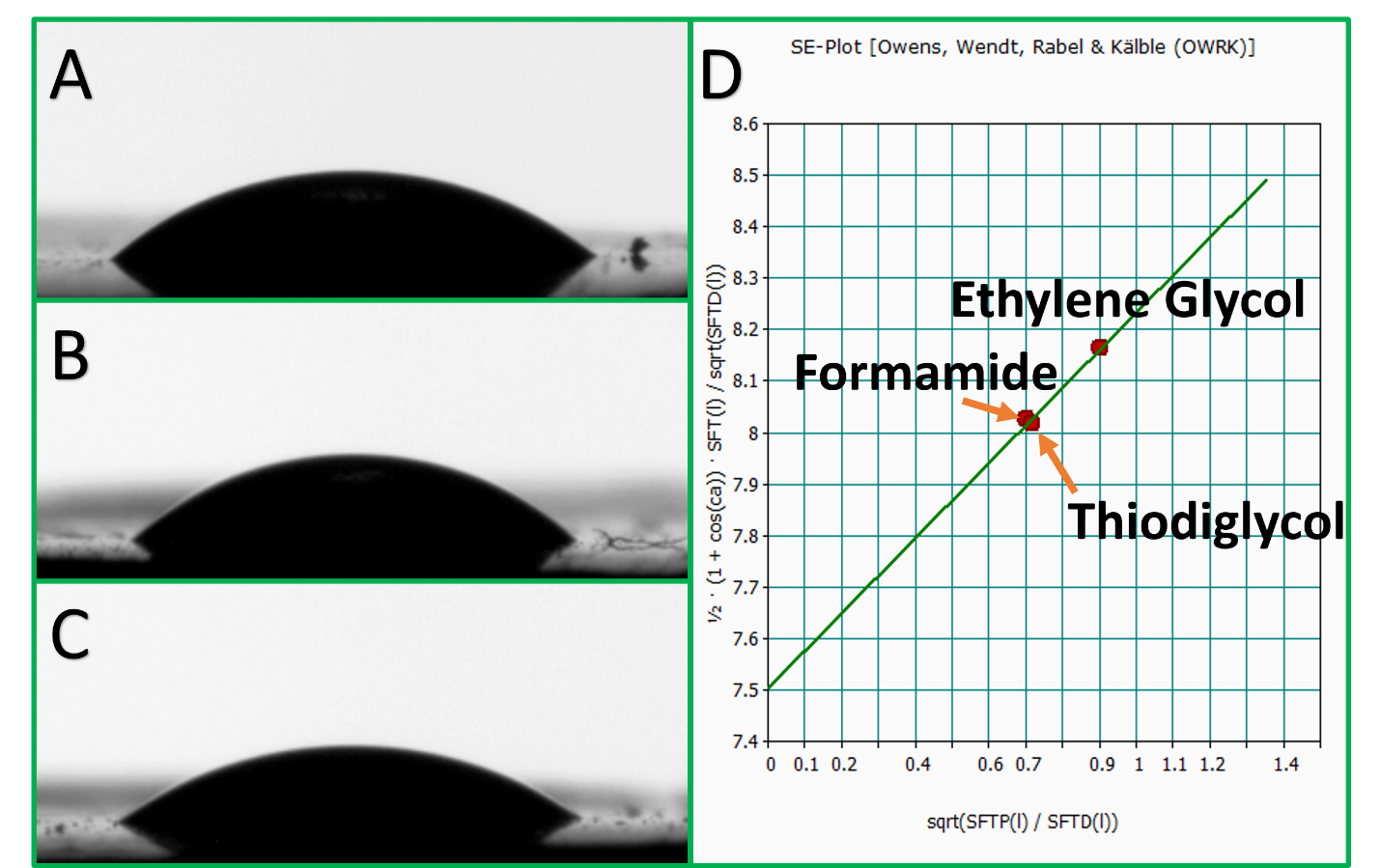


**Supplementary Figure 5 | Graphite Surface Energy Characterization.** Droplets formed by a) EG, b) formamide, and c) TDG on graphite to determine the contact angles needed for surface energy characterization for graphite. d) Surface energy plot for each of the probing liquids used to estimate the surface free energy components of graphite.

As previously mentioned, the surface energy values for LCO, PVDF, and graphite using the OWRK method were used to characterize the mixing interactions examined in the main article. A different surface energy calculation method was used for each of the electrode materials (LCO, PVDF, and graphite) to determine if the calculation method had a significant effect on the polar, dispersive, and total surface energy values. If there is a significant difference then the conclusions made in the main paper could be effected due to a possible change in the work of adhesion and work of cohesion calculations. Based on the Wu method, the polar, dispersive, and total surface energy values for each of the materials have very minor changes. The difference between the methods (OWRK and Wu) were small enough such that the conclusions made based on the work of adhesion and work of cohesion calculations would not have any change.

**Binder Distribution Difference between Dry Painted and Slurry-casted Electrodes**

Conventional and dry electrodes were cleaved and the cross section were examined by SEM. SEM micrographs were taken at different sections (top, middle and bottom part of the cross section, with the bottom section closest to the current collector). Within each section, SEM images were randomly taken at four locations to provide more data sampling. Representative micrographsare shown in Fig. 6S. It can be seen that slurry-cast electrodes feature more coverage on LCO particles while more exposed LCO particles are commonly seen in dry painted electrodes. As discussed in the main text, dry painted electrodes will have more LCO particles that are covered by loosely bonded binders which can be easily removed during sample cleavage. EDS spectra on selected LCO particles revealed more binders/carbons on individual LCO particle surface for slurry-casted electrodes. It is interesting to notice that in slurry-casted electrode, there are more binder/carbon coverage near the top section of the electrode, this phenomena will be studied more thoroughly in the future.


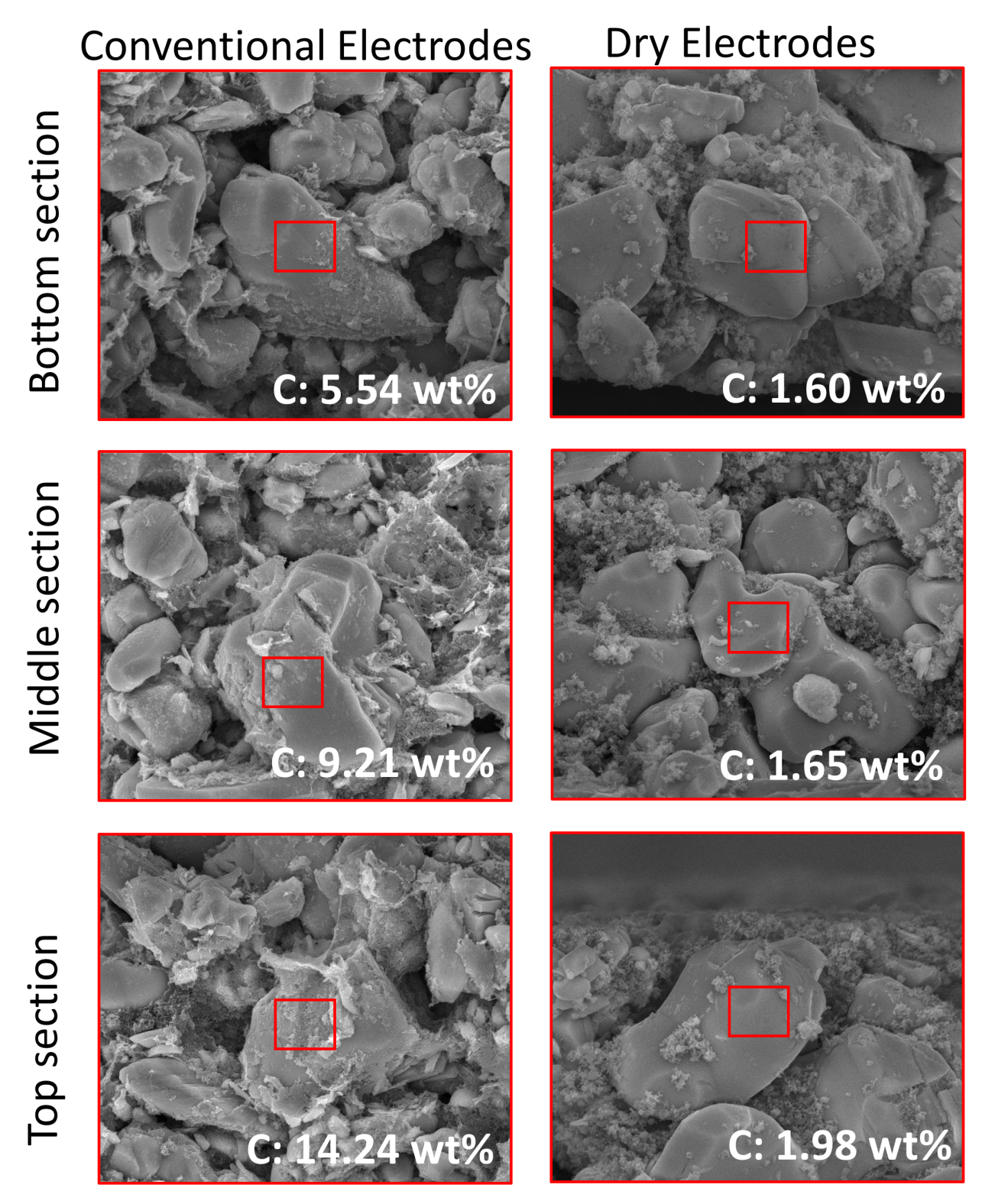


**E**

**F**

**D**

**C**

**B**

**A**

**Supplementary Figure 6 | SEM and EDS Characterizations of Binder/Carbon Coverage on LCO Particles.** SEM images of LCO particles in conventional processed electrode taken at the bottom a), middle c) and top e) section of the electrode. SEM images of LCO particles in dry processed electrode taken at the bottom b), middle d) and top e) section of the electrode. EDS spectra were taken at selected areas shown as red rectangles. The carbon weight percentages measured from EDS spectra are shown in each figure.

**Effect of Mixing Time**

Samples of 90% LiCoO_2_ and 10% PVDF was mixed with different mixing times to see the effect mixing time had on the distribution of PVDF throughout LiCoO_2_. 1 gram of the mixture was shaken vigorously by hand for 1 minute and sprayed. Subsequent mixing was performed with a BeadBug Microtube Homogenizer. For each mixing cycle with the homogenizer, 1 gram of material was mixed at a constant speed of 2,800 RPM. Mixing times with the homogenizer included 30 seconds, 3 minutes, and 90 minutes. Each of the four mixture types were sprayed with inlet pressure equal to 25 psi and a 1.5 in. gap distance between the current collector and electrode tip. The deposition characteristics were comparable for each of the samples but SEM micrographs were taken to determine the uniformity of PVDF particles throughout the surrounding LiCoO_2_ particles. Each of the samples showed a monolayer of PVDF forming on LiCoO_2_ particles. Supplementary Fig. 7S shows an SEM micrograph for a sample made after 3 minutes of mixing and for a sample made after 90 minutes of mixing. Therefore, it was determined that prolonged mixing of multiple materials yielded a negligible change in post-mixing material characteristics.


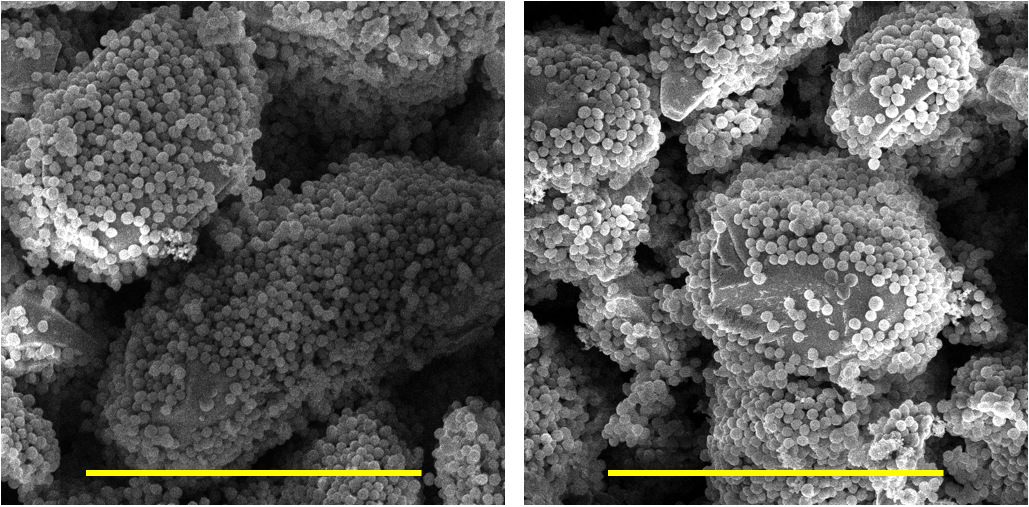


**Supplementary Figure 7** **| Effect of mixing time.** a) SEM micrograph of a sample made after 3 minutes of mixing time. b) SEM micrograph of a sample made after 90 minutes of mixing time. (scale bar represents 10 µm for both images)

**Effect of Spraying System Setup Parameters**

Obtaining a satisfactory deposition is dependent on the material type and also on the spraying system parameters. It should be noted that all samples were vertically sprayed as this would more easily show the differences in charging capabilities for the various materials. If a material is weakly charged, gravitational forces would be too large and the materials would not be drawn to the grounded current collector. A few system parameters can be changed to alter the depositions characteristics which include: charging voltage, carrier gas pressure, and the gap distance between the spraying gun electrode tip and the grounded current collector.

For this system, the charging voltage could be switched between 15 kV and 25 kV. Early results showed that the 15 kV charging voltage did not charge the material enough to be deposited onto the current collector. The 25 kV setting helped with charging the materials and was used for all tested samples.

Carrier gas pressure could range from almost 0 to a maximum of 25 psi. Generally, 25 psi gave the best deposition results but for some materials it was not ideal. A high carrier gas pressure did not work well for materials that did not charge and deposite very well. Since the particles did not charge, they were not drawn to the grounded current collector, therefore, the carrier gas would just blow the uncharged particles off of the current collector since there wasn’t any attraction between the particles and the current collector. For these materials the carrier gas pressure was reduced to 15 psi. Reducing the pressure even further would start to reduce the deposition quality again as the low pressure would not be enough to fluidize the particles well enough to make it to the spraying gun electrode to be charged, and then drawn to the current collector.

The gap distance between the spraying gun electrode tip was mostly held constant at 1.5 in. but in some cases needed to be changed depending on the material. Again, the gap had to be changed for materials that did not charge and deposit well at the standard 1.5 in. gap distance. For these weakly charged materials a 1.5 in. gap distance was too far of a distance to travel and deposit onto the current collector. The weakly charged particles was overcome by the stronger gravitational forces and failed to deposit onto the current collector. A shorter gap distance helped with this as it reduced the distance the particles needed to travel for deposition. But just like lowering the carrier gas by too much, if the gap distance is short it will start to give an unsatisfactory deposition. The short gap distance will cause the coated area to be too small as the material doesn’t have enough distance to spread out and create a large enough coating for mechanical or electrochemical tests.

**Control of Electrode Thickness**

A mixture of 80% LiCoO_2_ , 10% C65, and 10% PVDF was used to determine how the amount of material loaded to the spraying bottle changed the electrode thickness. Material amounts ranged from 0.5 g, 1.0 g, 2.0 g, and 7.5 g. Each of the four material amounts were sprayed with inlet pressure equal to 25 psi and a 1.5 in. gap distance between the current collector and electrode tip. Before and after spraying, the current collector was weighed so that the amount of material deposited would be known. With the amount of material and density of each of the materials known, the thickness of the coated electrode could be estimated after measuring the area of the coated electrode. It can be seen in Supplementary Fig. 6 that the thickness gradually increases when the amount of material loaded into the spray bottle increases. Based on the results, 1.0 g of material was used for subsequent depositions as this amount of material doens’t require much time to mix and it will still allow for an electrode thickness of 55-60 µm. It should be noted that 1.0 g of each material used for Co-Spray 2 tests instead of 1.0 total grams between the two materials.


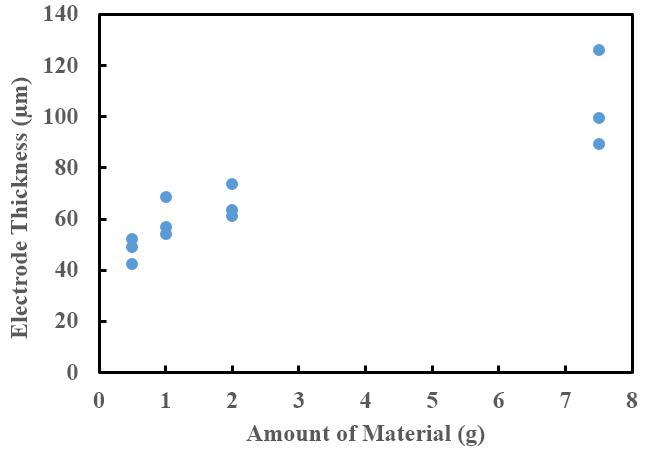


Supplementary Figure 8 | Amount of material loaded before deposition vs. thickness of the sprayed electrode.

**References**

[1] Dixit, U.S., Narayayanan, R.G., in *Metal Forming: Technology and Process Modelling*, Ch. 1 (McGraw Hill Education Private Limited, New Delhi, 2013)

[2] Park, S.H, et al., Synthesis and structural characterization of layered Li[Ni_1/3_Co_1/3_Mn_1/3_]O_2_ cathode materials by ultrasonic spray pyrolysis method. *Electrochim. Acta* **49**, 557-563 (2004).

[3] Choi, J., Manthiram, A., Investigation of the Irreversible Capacity Loss in the Layered LiNi_1/3_Mn_1/3_Co_1/3_O_2_ Cathodes. *Electrochem. Solid-State Lett*. **8** C102-C105 (2005).

[4] Nowak, E.; Combes, G., Stitt, E.H. & Pacek, A.W. A comparison of contact angle measurement techniques applied to highly porous catalyst supports. *Powder Technol.* **233**, 52-64 (2013)
